# Design and Testing of a Simulator for Micro-Vibration Testing of Star Sensor

**DOI:** 10.3390/mi14091652

**Published:** 2023-08-22

**Authors:** He Zhu, Shuai He, Xiaoming Wang, Chao Qin, Lin Li, Xiangyang Sun

**Affiliations:** 1 Changchun Institute of Optics, Fine Mechanics and Physics, Chinese Academy of Sciences, Changchun 130033, China; zhuhe19@mails.ucas.ac.cn (H.Z.); heshuai@ciomp.ac.cn (S.H.); qinchao@ciomp.ac.cn (C.Q.); sunxiangyang19@mails.ucas.ac.cn (X.S.); 2 University of Chinese Academy of Sciences, Beijing 100049, China; 3 Beijing Institute of Control Engineering, Beijing 100049, China; 4 China Academy of Space Technology, Beijing 100049, China

**Keywords:** space micro-vibration, acceleration simulator, gravity unloading system, star sensor

## Abstract

(1) Background: To simulate the micro-vibration environment of the star sensor mounting surface, a multi-dimensional micro-vibration simulator based on the Gough–Stewart platform was designed, which could effectively reproduce space six-dimensional acceleration; (2) Methods: Firstly, the integrated design of a gravity unloading system and micro-vibration simulation platform was adopted, and the first six natural frequencies and mode diagrams of the simulator were obtained by modal analysis. Then, the complete dynamic equation of the simulator was established, and the relationship between the acceleration of the upper platform and the driving force of the legs was deduced, which was verified by co-simulation. Finally, the whole machine test was carried out using the frequency response function based on the actual simulator without multiple iterations; (3) Results: The test results show that the micro-vibration simulator can reproduce space six-dimensional acceleration, with an output bandwidth of 5–300 Hz, and maximum error of 9.19%; (4) Conclusions: The micro-vibration simulator platform has the characteristics of a highly precise, large analog bandwidth and takes up less space, is conducive to transportation, and can accurately reproduce the six-degree-of-freedom space micro-vibrations for the star sensor.

## 1. Introduction

Star sensors possess a high attitude measurement accuracy, robust autonomous navigation capability, and remarkable anti-interference ability, thus playing a pivotal role in determining the accurate attitude and control of a spacecraft [1,2,3,4]. During the on-orbit operation of spacecraft, space micro-vibrations can be generated by on-board motion equipment and devices in spacecraft, such as reaction/momentum wheel assemblies, cryo-coolers, and solar array drive mechanisms, which can severely degrade the imaging quality of the star sensor and even prevent the proper extraction of the star map [5,6,7,8]. In order to enhance the performance of the star sensor, it is essential to analyze the impact of micro-vibrations on its imaging alongside its ability to reproduce spatial micro-vibrations, which was a prerequisite for carrying out this work.

At present, scholars have conducted extensive research on space micro-vibration simulation platforms. Hostens et al. [9,10] developed a hydraulic-driven six-degree-of-freedom vibration stage that has been primarily utilized for conducting vibration tests on large-scale mechanical equipment due to its ability to generate high magnitudes of vibration. Park et al. [11,12] introduced two successive generations of vibration simulators aimed at conducting jitter tests for satellite development and evaluating the performance of isolators. Xu et al. [13] and Wang et al. [14] proposed the disturbance force simulator and the acceleration simulator, which can be applied in ground experiments as a vibration source device. However, the above micro-vibration simulation platform is not specific to any particular aerospace component. This article aimed to design a dedicated micro-vibration simulator for star sensors to analyze the operational performance of star sensors under micro-vibration in space.

When considering the gravity unloading of the motion platform and star sensors in the simulator, practicality, spatial occupancy, and the operation life of the gravity unloading mechanism need to be taken into account. There are several gravity unloading methods available for the ground testing of aerospace products, including air flotation [15,16], magnetic levitation [17], suspension methods, and so on. The air flotation-based gravity unloading method has been applied in the aerospace industry since its early days. However, it can introduce additional forces and moments to the mechanism to be unloaded due to issues such as machining, assembly, and variations in the air film [18]. Magnetic levitation gravity compensation has advantages such as no mechanical friction and a high support force. However, electromagnetic effects can introduce power-frequency interference to micro-vibration simulation. The suspension method is widely used: Alessandro et al. [19] utilized suspension ropes to achieve gravity unloading for a triangular bracket and four dummy reaction wheels during the micro-vibration mitigation platform testing. Chen et al. [20] employed a low-frequency suspension system to simulate an on-orbit environment to investigate the impact of micro-vibrations on image displacement. Li et al. [21] and Ma et al. [22], respectively, designed a suspension system to achieve the gravity unloading of the payloads to imitate the on-orbit microgravity environment. However, the suspension method has drawbacks such as large spatial occupation, inconvenience in transportation, and difficulties in reassembly and adjustment. In the previous designs of micro-vibration simulators, a method involving the use of self-springing in excitation legs for gravity unloading has been employed [23]. However, this method is only suitable for cases where the motion platform and load have a small mass.

There are two main approaches to enhancing the performance of micro-vibration simulators. The first approach is to optimize the structural components to minimize the impact of nonlinearity on the test results. The second approach is to employ advanced control strategies to improve the performance of the simulator. The control strategy of the parallel manipulator can be divided into two types [24,25]: hinge space control and task space control. The hinge space control method neglects the dynamic characteristics of the parallel manipulator. The task space control method is currently a developing trend; however, it has poor robustness. In order to overcome this drawback, Yang et al. [26] designed a robust PI controller considering the effects of uncertainties for the acceleration control of the micro-vibration simulator, while it also required high-precision feedback displacement, and the testing process is difficult. The main form of micro-vibration is the multi-frequency line spectrum [27]; the simulation platform is only required to reproduce the vibration of a multi-frequency line spectrum, and trajectory tracking does not need to be achieved. Thus, Zhu et al. [23] simplified the control strategy and proposed a frequency response function control method based on the dynamics model. However, this method relies on a theoretical model with a low control accuracy and requires several iterations.

Accordingly, this paper proposes a micro-vibration simulator based on the Gough–Stewart platform, which is capable of reproducing multi-dimensional accelerations with varied frequency spectrum characteristics. The previous simulator has not been utilized for actual space products, whereas the simulator in this paper was specifically designed to provide six-dimensional space micro-vibrations for the star sensor. To design a set of multi-dimensional micro-vibration simulators integrated with the gravity unloading mechanism, space occupation, and portability need to be considered. Furthermore, it has the characteristics of a larger frequency bandwidth and higher precision than the simulator of similar dimensions proposed by previous authors.

This paper is organized as follows: Section 2 presents the structure of the integrated platform of the gravity unloading mechanism and micro-vibration simulator, and modal analysis is used to obtain the first six-order radical frequency of the platform. In Section 3, the full dynamic equations of the simulation platform are established, and the correlation between the acceleration of the upper platform and the excitation force of the legs is derived and validated through co-simulation. In Section 4, a control method based on the frequency response is employed for the whole machine test, and it can be seen that the performance of the simulator was significantly enhanced in terms of frequency bandwidth and precision. Section 5 draws conclusions about the performance of the simulator.

## 2. Structural Design and Simulation of Simulator

**Figure 1:**
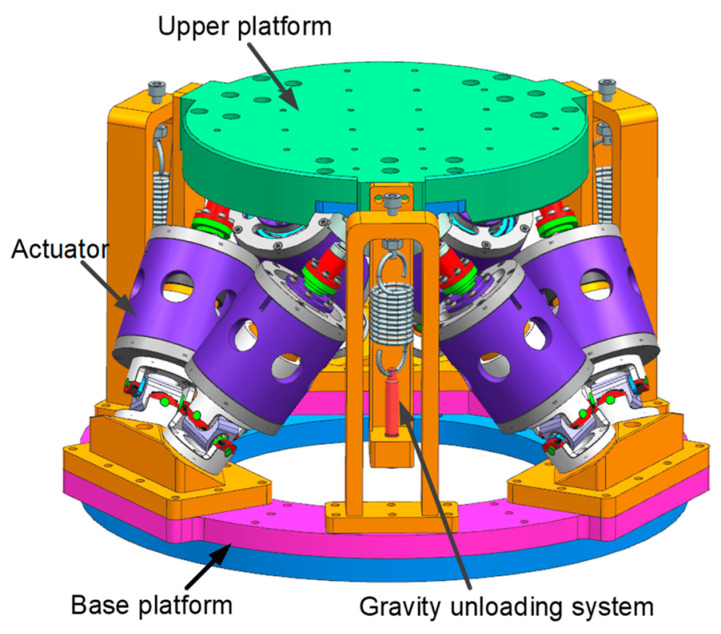
The structural diagram of multidimensional micro-vibration simulator.

The detailed structure of the multi-dimensional micro-vibration simulator based on the Gough–Steward configuration is depicted in Figure 1. The simulator in this paper was especially used for the micro-vibration test of the star sensor where the previous large suspension system would occupy space and impede the use of the star sensor; thus, an integrated design of the gravity unloading system and micro-vibration simulation platform was adopted. Three sets of gravity unloading mechanisms were evenly distributed and installed on the micro-vibration simulation platform to achieve gravity unloading in a confined space.

### 2.1. Structure Design

**Figure 2:**
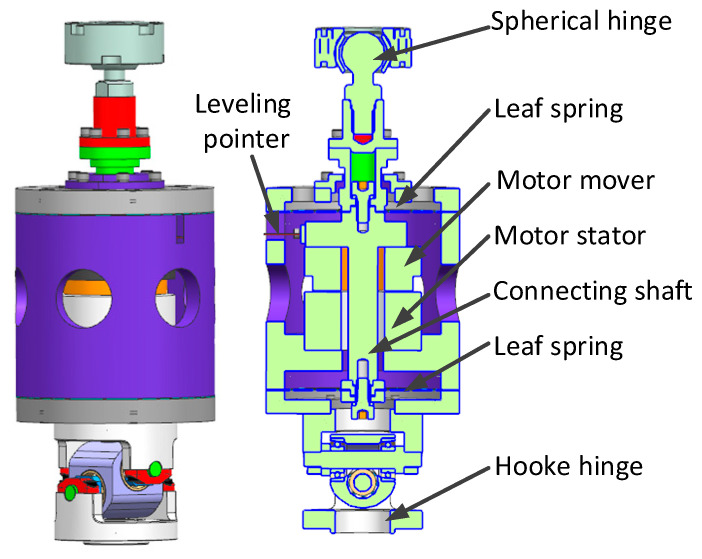
The structure diagram of single-axis actuator.

The simulator consists of the upper platform assembly, the base platform assembly, six actuators, and a gravity unloading system. The upper platform assembly consists of an upper platform and a ball-hinged connection block on which the star-sensitive device is mounted. The base platform assembly consists of a base platform, base platform adapter plate, and Hooker hinge connection block, which serves as the mounting and carrying platform. Each actuator mainly consists of a ball hinge, a connecting shaft, two spring plates, the voice coil motor, an envelope, and a Hooker hinge. The connecting shaft is supported by the upper and lower spring plates, with the motor mover fixed to the connecting shaft and the stator and envelope secured in place, as shown in Figure 2. Two spring plates mounted in parallel on the connecting shaft can serve a similar purpose to linear bearings, thus restricting the exciter moving parts to an axial motion only.

**Figure 3:**
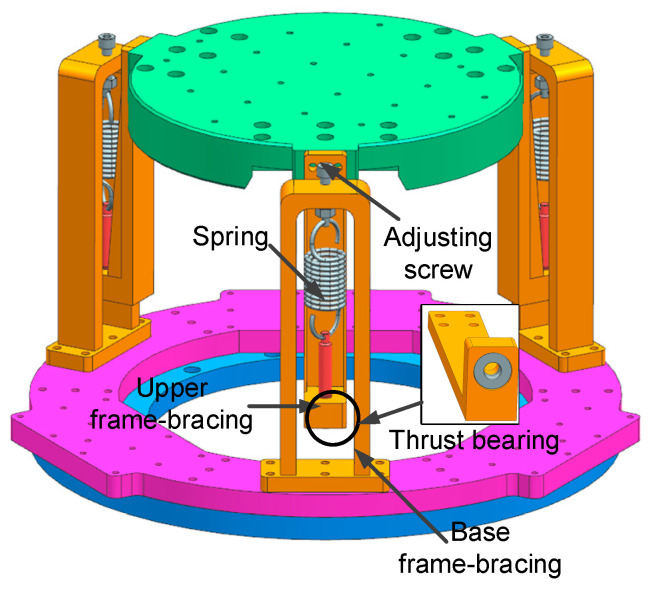
The gravity unloading system.

The detailed structure of the gravity unloading system is depicted in Figure 3, which consists of the upper and lower support frames, springs, adjusting screws, and thrust bearings to unload the gravity of the simulator’s moving parts and loads. The unloading spring is the core component of the system, providing the tension necessary for the gravity unloading of the micro-vibration simulator. The length of the spring is adjusted by means of an adjusting nut at the upper end and at the lower end; the spring is connected to a thrust bearing to release the torque from the rotating screw, thus leveling and returning the upper platform to zero. The integration of the gravity unloading system with the simulator allows the micro-vibration simulation platform to take up less space, be more adaptable to the environment and facilitate transportation.

In summary, the final micro-vibration simulation platform has an upper platform surface size of φ300 mm, an overall lower platform surface size of φ450 mm, an overall height of 288 mm, and a mass of 35 kg.

### 2.2. Simulation Analysis

**Figure 4:**
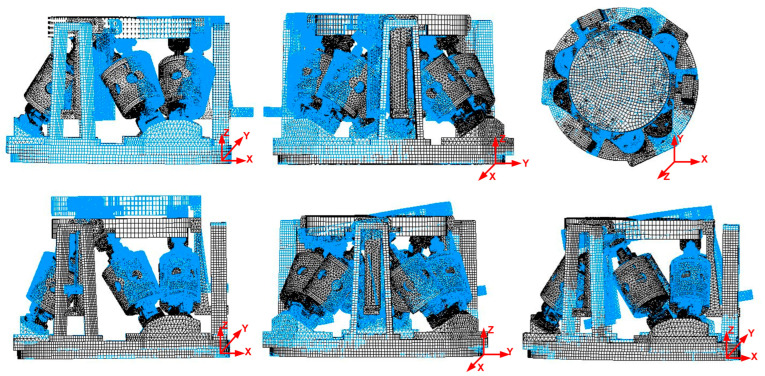
Cloud images of the first six-order modes.

After completing the design of the platform, the finite element model of the six-degree-of-freedom micro-vibration simulation platform was established, and the modal analysis was carried out. The first six-order modes are shown in Table 1, and cloud images are shown in Figure 4. It can be seen that the first six modes of the platform were within 5 Hz. In order to avoid the resonance peak of the system, the acceleration frequency should be set above 5 Hz in the subsequent micro-vibration simulation.

## 3. Dynamic Model and Validation

### 3.1. Complete Dynamic Equation

**Figure 5:**
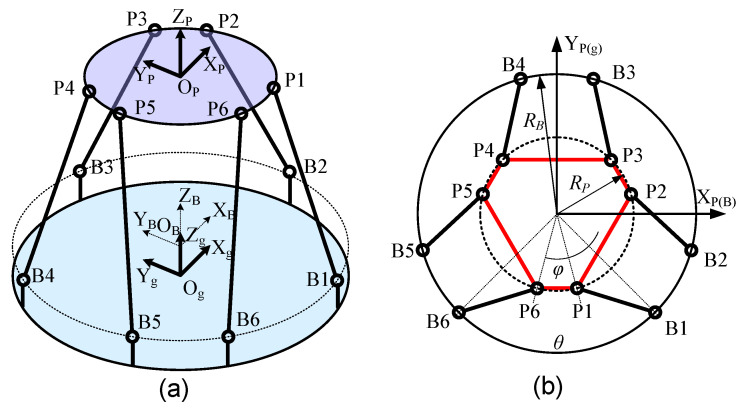
The structural diagram of micro-vibration simulation platform: (**a**) Axonometric view; (**b**) Vertical view.

The structural diagram of the simulation platform is shown in Figure 5. The general elastic force of the six legs subjected to the spring plate is:

(1) $$f_{e}=-k\cdot J\cdot q$$

where $f_{e}$ is a 6 × 1 vector matrix of the elastic forces on each leg, *k* denotes the axial stiffness coefficient, q is the general velocity of the moving platform, $q={\left[x\;y\;z\;\gamma \;\beta \;\alpha \right]}^{T}$, and J is the actuator Jacobian matrix expressing mappings from the general velocity to the actuator sliding velocities.

Similarly, the general damping force $f_{c}$ can be described as:

(2) $$f_{c}=-c\cdot J\cdot \dot{q}$$

where $f_{c}$ is a 6 × 1 matrix and *c* is the damping coefficient of the actuator.

Considering the applied generalized force exerted on the moving platform **Γ** and the actuator forces supposed by the voice coil motor **F**, the equilibrium equation could be obtained as follows:

(3) $$\Gamma =J^{T}\left(f_{e}^{}+f_{c}^{}+F\right)$$

where $F={\left[\begin{array}{llllll} f_{1} & f_{2} & f_{3} & f_{4} & f_{5} & f_{6} \end{array}\right]}^{T}$, $\Gamma ={\left[\begin{array}{llllll} \tau_{1} & \tau_{2} & \tau_{3} & \tau_{4} & \tau_{5} & \tau_{6} \end{array}\right]}^{T}$.

The dynamic model for the upper platform as a rigid body ignoring the inertia of the actuators could be derived using the Newton–Euler method:

(4) m⋅p¨cB=Γ3×1

(5) RPB⋅IP⋅RTPB⋅ω˙+ω˜⋅RPB⋅PI⋅RPBT⋅ω+m⋅RPB⋅pcP×t¨=Γ4×6

where *m* is the payload mass, p¨cB denotes the acceleration vector of the centroid on the moving platform and load under the base frame {*B*}, pcP denotes the position vector of the centroid on the moving platform and load under the body frame {*P*}, $\dot{\omega }$ are the angular acceleration of the moving platform, $\tilde{\omega }$ is a skew-symmetric matrix of ω, and RPB is the rotation matrix of the transformation from the body frame {*P*} to the base frame {*B*}. ${}^{P}I$ is the inertia matrix with respect to the frame {*P*}, $\Gamma_{3\times 1}=\left[\tau_{1},\tau_{2},\tau_{3}\right]$, $\Gamma_{4\times 6}=\left[\tau_{4},\tau_{5},\tau_{6}\right]$.

(4) and (5) can be written in matrix form:

(6) mE3m⋅RPB⋅p˜cTP⋅RPBTm⋅RPB⋅p˜cP⋅RPBTRPB⋅IP⋅RTPBq¨+000ω˜RPB⋅IP⋅RTPB+c⋅JTJq˙+mE30ω˜2⋅RPB⋅p˜cP+k⋅JT⋅J⋅q=JTF

Considering the inertia of the actuators, (6) can be written as:

(7) mE3m⋅RPB⋅p˜cTP⋅RTPBm⋅RPB⋅p˜cP⋅RPBTRPB⋅PI⋅RPBTq¨+000ω˜RPB⋅PI⋅RpBT+c⋅JTJq˙+mE30ω˜2⋅RPB⋅p˜cP+k⋅JT⋅J⋅q=−HpFp

where expressions for $H_{P}$ and $F_{P}$ are given by:

HP=E3E3E3E3E3E3RPBPp˜1RPBTRPBPp˜2RPBTRPBPp˜3RPBTRPBPp˜4RPBTRPBPp˜5RPBTRPBPp˜6RPBT

$$F_{P}={\left[\begin{array}{llllll} f_{p1}^{T} & f_{p2}^{T} & f_{p3}^{T} & f_{p4}^{T} & f_{p5}^{T} & f_{p6}^{T} \end{array}\right]}^{T}$$

Then, the complete dynamic equation of the vibration simulator can be described as:

(8) $$M\left(q\right)\ddot{q}+C(q,\dot{q})\dot{q}+K(q)q=J^{T}F$$

where $M\left(q\right)$ is a 6 × 6 mass matrix, $C(q,\dot{q})$ is a 6 × 6 matrix of the centrifugal and Coriolis force terms, K(q) is a 6 × 6 matrix of the generalized stiffness, and *F* is a 6 × 1 vector representing actuator forces.

Pre-multiplying both sides of (8) with $J^{-T}$, the actuator forces are given by:

(9) $$F=J^{-T}[M\left(q\right)\ddot{q}+C(q,\dot{q})\dot{q}+K(q)q]$$

### 3.2. Co-Simulation Verification

**Figure 6:**
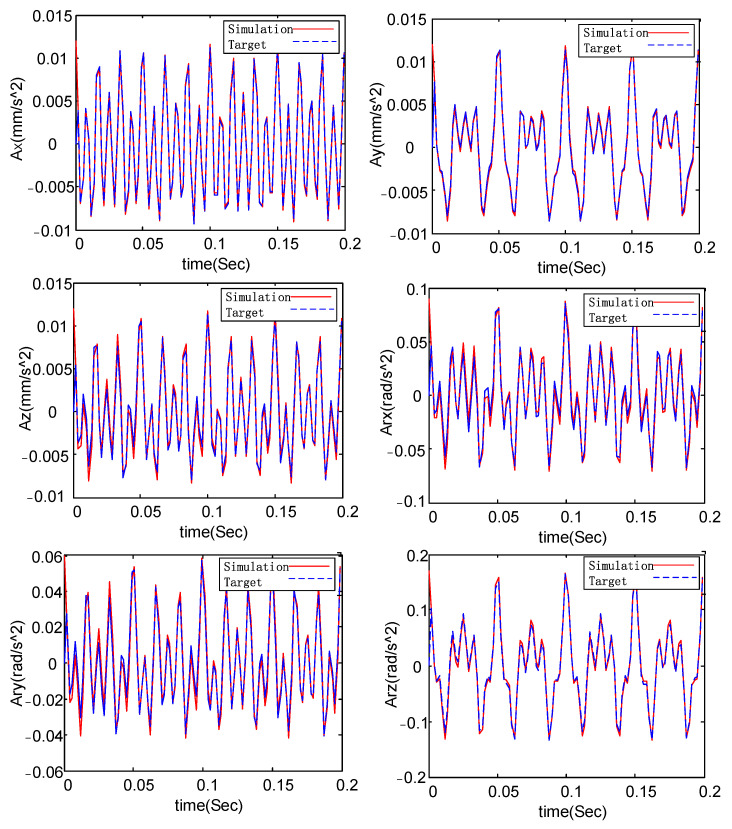
The structural diagram of micro-vibration simulation platform.

To verify the validity of the simulator’s dynamic equation, a co-simulation was carried out. The specific process is as follows: The target acceleration values in Table 2 are input, and the excitation force of each driving leg is calculated using Equation (9). The excitation force is then used to drive the model in the virtual prototype, resulting in the simulation value of the upper platform acceleration, as illustrated in Figure 6. As seen in Figure 6, the simulated acceleration curves are quite consistent with the target–acceleration curves, thus verifying the accuracy of the dynamics model for the micro-vibration simulation platform.

## 4. Experimental Section

### 4.1. Control Strategy

Because the simulation platform was only required to reproduce the vibration of a multi-frequency line spectrum, which is the main form of micro-vibration, and trajectory tracking did not need to be achieved, the control method required could then be simplified. The uncertainty over the parameter values (e.g., the payload mass, the inertial tensor, and the stiffness of the springs) meant that the theoretical model of an actual micro-vibration platform would not coincide exactly with the actual system. The micro-vibration of the multi-frequency line spectrum could be controlled using the frequency response function. If testing was performed directly using the frequency response function based on the dynamic model, the control accuracy was low and multiple iterations were required.

Therefore, this paper adopts a control method that involves extracting transfer functions from the actual simulator. This approach eliminates the reliance on theoretical models and provides excellent control accuracy. The specific procedure is outlined below:

First, sinusoidal excitation forces are applied individually to the six legs of the micro-vibration simulator as follows:

(10) $$F(j\omega )={\left[fe^{j\omega }\;0\;0\;0\;0\;0\right]}^{\prime }$$

The six-dimensional acceleration measured at the center point of the upper platform is:

(11) $$A(j\omega )=e^{j\omega }{\left[a_{11}e^{j\varphi_{11}}\;a_{21}e^{j\varphi_{21}}\;a_{31}e^{j\varphi_{31}}\;a_{41}e^{j\varphi_{41}}\;a_{51}e^{j\varphi_{51}}\;a_{61}e^{j\varphi_{61}}\right]}^{\prime }$$

The transfer function $H_{1}(\omega )$ of the acceleration response of the load platform relative to the driving force of actuator 1 is:

(12) $$\begin{array}{cc} H_{1}(\omega ) & ={\left[H_{11}\;H_{12}\;H_{13}\;H_{14}\;H_{15}\;H_{16}\right]}^{\prime }\\ & =\frac{{\left[a_{11}e^{j\varphi_{11}},a_{21}e^{j\varphi_{21}},a_{31}e^{j\varphi_{31}},a_{41}e^{j\varphi_{41}},a_{51}e^{j\varphi_{51}},a_{61}e^{j\varphi_{61}}\right]}^{\prime }}{f} \end{array}$$

Similarly, by applying $F={\left[0\;fe^{j\omega }\;0\;0\;0\;0\right]}^{\prime }$, $F={\left[0\;0\;fe^{j\omega }\;0\;0\;0\right]}^{\prime }$,$F={\left[0\;0\;0\;fe^{j\omega }\;0\;0\right]}^{\prime }$, $F={\left[0\;0\;0\;0\;fe^{j\omega }\;0\right]}^{\prime }$, and $F={\left[0\;0\;0\;0\;0\;fe^{j\omega }\right]}^{\prime }$, respectively, the 6-d of acceleration at the center point of the upper platform under the corresponding actuator force can be obtained, denoted as $A_{2}$, $A_{3}$, $A_{4}$, $A_{5}$, $A_{6}$.

Thus, the frequency response function at frequency ω can be obtained:

(13) $$H\left(\omega \right)=\left[A_{1},A_{2},A_{3},A_{4},A_{5},A_{6}\right]/fe^{j\omega }=\left[\begin{array}{c} H_{11}\;H_{12}\;H_{13}\;H_{14}\;H_{15}\;H_{16}\\ H_{21}\;H_{22}\;H_{23}\;H_{24}\;H_{25}\;H_{26}\\ H_{31}\;H_{32}\;H_{33}\;H_{34}\;H_{35}\;H_{36}\\ H_{41}\;H_{42}\;H_{43}\;H_{44}\;H_{45}\;H_{46}\\ H_{51}\;H_{52}\;H_{53}\;H_{54}\;H_{55}\;H_{56}\\ H_{61}\;H_{62}\;H_{63}\;H_{64}\;H_{65}\;H_{66} \end{array}\right]$$

The initial forces $F_{0}\left(\omega_{i}\right)$ are then obtained:

(14) $$F_{0}\left(\omega_{i}\right)=H\left(\omega \right)A\left(\omega_{i}\right)$$

where $F_{j}(\omega_{i})$ is the control forces at step *j*, $A_{j}(\omega_{i})$ is the actual output accelerations at step *j*, $A(\omega_{i})$ is the desired accelerations. The deviation of the actual output accelerations from the desired accelerations at step *j* are: $e_{j}(\omega_{i})=A_{j}(\omega_{i})-A(\omega_{i})$. The control input forces for step *j* + 1 are then updated using an iterative control penalty as follows:

(15) $$F_{j+1}\left(\omega_{i}\right)=F_{j}\left(\omega_{i}\right)-K_{j}\left(\omega_{i}\right)H\left(\omega_{i}\right)e_{j}\left(\omega_{i}\right)$$

where the controller gain $K_{j}(\omega_{i})$ can be set at 1.0.

### 4.2. 6-DOF Micro-Vibration Test

**Figure 7:**
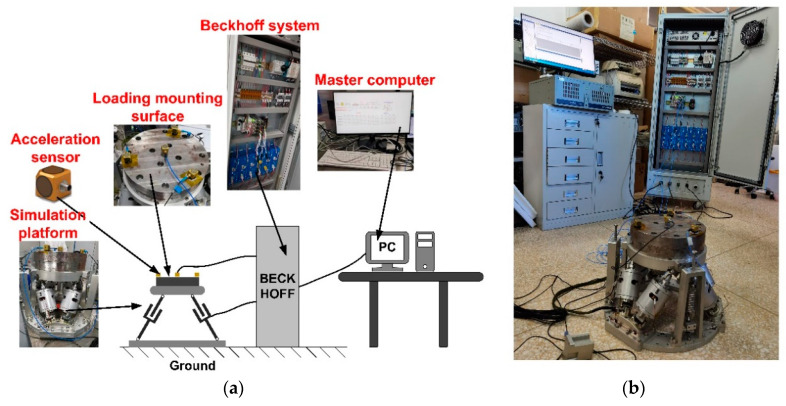
The whole machine test system diagram. (**a**) illustration of micro-vibration test; (**b**) the real test system.

**Figure 8:**
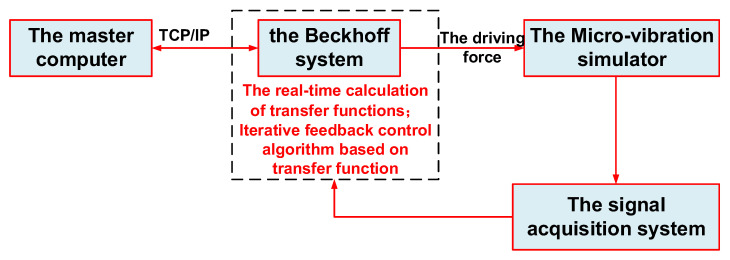
The flow chart of the testing system.

The six-degree-of-freedom micro-vibration simulation platform provides the space micro-vibration environment for the star sensor, but due to project scheduling and confidentiality considerations, a 35 kg payload was used instead of the star-sensitive device for the micro-vibration test in this paper. The test system is depicted in Figure 7, comprising a micro-vibration simulator, the Beckhoff control system, the signal acquisition system, and the master computer. The master computer is mainly responsible for running the operation interface and sending instructions. The Beckhoff control system is responsible for the real-time calculation of transfer functions and control algorithms and for generating the driving force. The micro-vibration simulator generates the expected acceleration according to the driving force. The signal acquisition system collects the accelerations of the simulation platform. The test procedure is as follows: the target accelerations are input at the master computer and are then converted by the Beckhoff control system into the corresponding force to drive the legs of the simulator. The data acquisition module of the Beckhoff system collects the values of acceleration sensors and feeds them back to the master computer. The flow chart of the testing system is shown in Figure 8.

**Figure 9:**
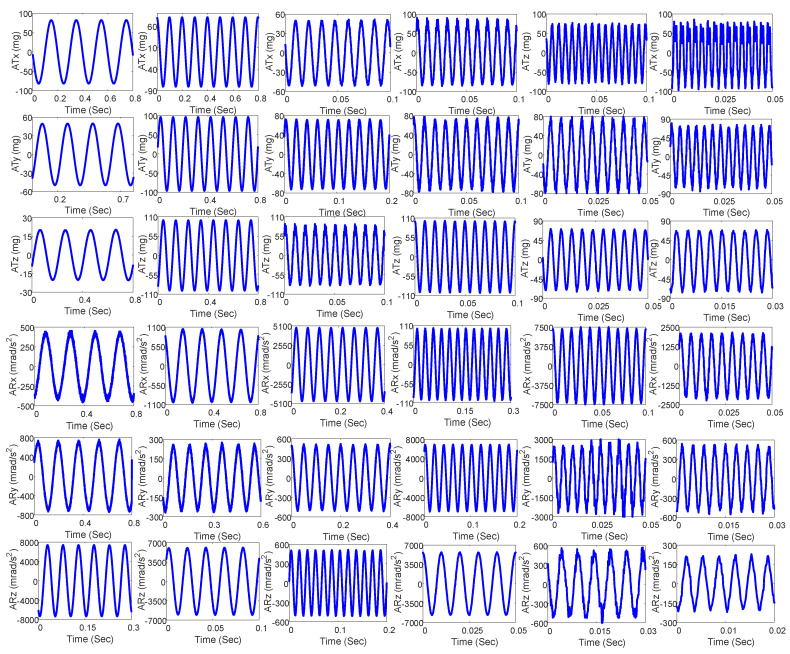
The single-frequency test results in time domain.

**Figure 10:**
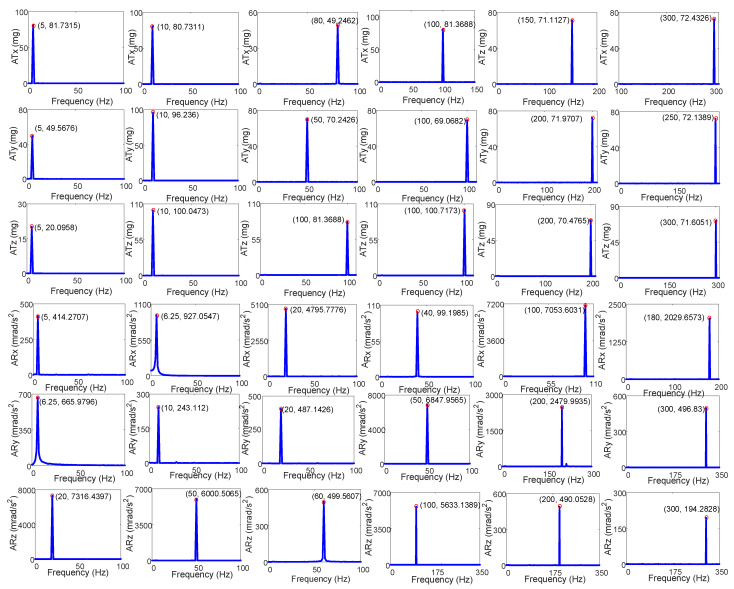
The single-frequency test results in frequency domain.

**Figure 11:**
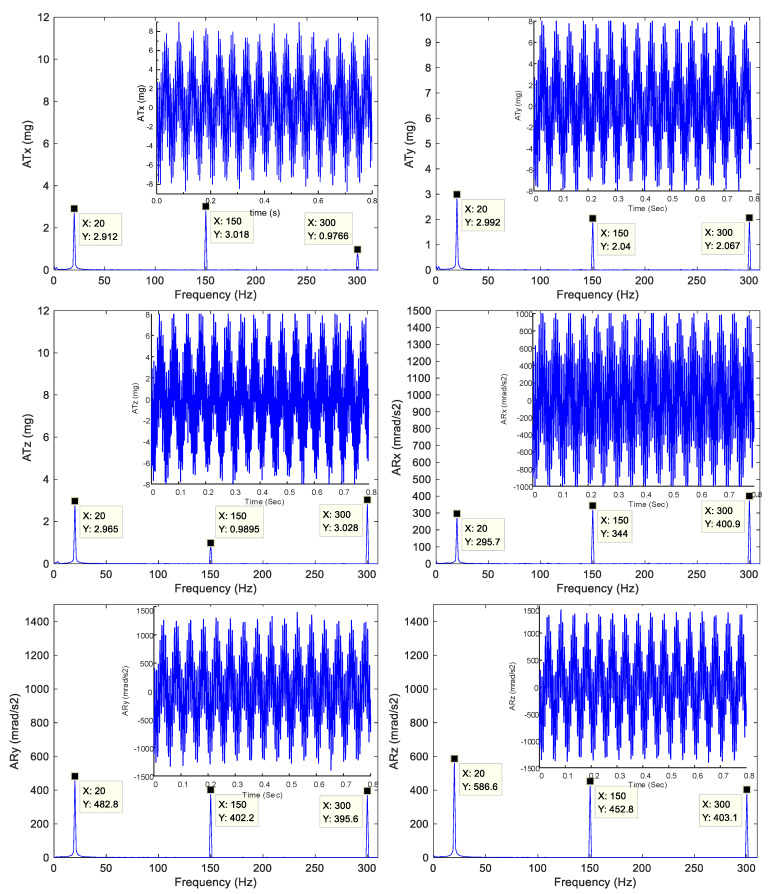
Multi-frequency micro-vibration experimental curve.

The simulation platform was tested at different frequencies and magnitudes in six directions, with some of the test results presented in Table 3, Table 4 and Table 5. Table 3 displays the single-frequency translation test results, Table 4 displays the single-frequency rotation test results, Figure 9 illustrates the single-frequency actual acceleration curves in Table 3 and Table 4 in the time domain, and Figure 10 displays the corresponding test curves in the frequency domain where $ATx_{}$, ATy and ATz are the three translational acceleration trajectories at the upper platform center point in units of mg and ARx, ARy and ARz are the three angular acceleration trajectories at the upper platform center point in units of mrad/s^2^. It can be seen from the figures and tables that the maximum amplitude error of the single-frequency translational test was 9.19%, appearing in the X-axis at 5 Hz, and the maximum amplitude error of the single-frequency rotational test was 7.30%, appearing on the X-axis at 6 Hz. The maximum translational acceleration output magnitude was 100.71 mg, which appeared on the Z-axis at 100 Hz, and the maximum rotational acceleration output magnitude was 7316.44 mrad/s^2^ appearing on the Z-axis at 20 Hz. Table 5 displays the multi-frequency test conditions, results, and errors. Figure 11 presents the corresponding actual acceleration curves in six directions in the time and frequency domain. The maximum error of the multi-frequency test was 3.5% appearing in the Y-axis at a translational acceleration of 300 Hz.

Overall, the magnitude error is relatively small; thus, the initial excitation force is directly utilized to drive the legs without iterating on it using Equation (15) in the actual testing. The results demonstrate that the micro-vibration simulator proposed in this paper can accurately reproduce six-degree-of-freedom space micro-vibrations and is highly precise; thus, it can be employed as a micro-vibration generator for the star sensor.

## 5. Conclusions

This study presents the structural design, dynamics modeling, and the experiment of a micro-vibration simulator, which can reproduce 6-DOF micro-vibrations with different amplitudes and frequencies. An integrated design of a gravity unloading system and micro-vibration simulation platform was adopted, and the first six-order radical frequency of the platform was within 5 Hz during the modal analysis. This dynamics model was derived, and a co-simulation was used to verify the validity of the dynamics model. The transfer function was used to control the simulator, and the experimental results show that the output bandwidth of the simulator was 5–300 Hz, the maximum error of magnitude was 9.19%, and the excitation force need not be iterated. This demonstrates that the micro-vibration simulator proposed in this paper is highly precise and can accurately generate the six-degree-of-freedom space micro-vibrations for star sensors.

## Figures and Tables

**Table 1:** First six-order modes of micro-vibration simulation platform.

Order	Frequency (Hz)	Description of Vibration Pattern
1	1.6473	Translation in the X-axis direction
2	1.6473	Translation in the Y-axis direction
3	2.3519	Rotation in the Z-axis direction
4	3.7542	Translation in the Z-axis direction
5	4.1668	Rotation in the X-axis direction
6	4.1669	Rotation in the Y-axis direction

**Table 2:** Target acceleration of upper platform.

Frequency (Hz)	*A_x_* (mm∙s^−2^)	*A_y_* (mm∙s^−2^)	*A_z_* (mm∙s^−2^)	*A_rx_* (rad∙s^−2^)	*A_ry_* (rad∙s^−2^)	*A_rz_* (rad∙s^−2^)
40	1.0	5.0	2.0	0.03	0.01	0.08
60	3.0	4.0	4.0	0.02	0.02	0.04
120	8.0	3.0	6.0	0.04	0.03	0.05

**Table 3:** Single-frequency translational acceleration test.

Direction	Frequency (Hz)	Target Acceleration (mg)	Actual Acceleration (mg)	Magnitude Error (%)
x	5	90	81.73	9.19
x	10	80	80.73	0.91
x	80	50	49.25	1.50
x	100	80	81.37	1.71
x	150	70	71.11	1.59
x	300	70	72.43	3.47
y	5	50	49.57	0.86
y	10	95	96.24	1.31
y	50	70	70.24	0.34
y	100	70	69.07	1.33
y	200	70	71.97	2.81
y	250	70	72.14	3.06
z	5	20	20.10	0.50
z	10	100	100.05	0.05
z	100	80	81.37	1.71
z	100	100	100.71	0.71
z	250	70	70.48	0.69
z	300	70	71.61	2.30

**Table 4:** Single-frequency rotational acceleration test.

Direction	Frequency (Hz)	Target Acceleration (mrad/s^2^)	Actual Acceleration (mrad/s^2^)	Magnitude Error (%)
rx	5	400	414.27	3.57
rx	6	1000	927.05	7.30
rx	20	5000	4975.78	0.48
rx	40	100	99.20	0.8
rx	100	7000	7053.60	0.77
rx	180	2000	2029.66	1.48
ry	6	700	665.98	4.86
ry	10	250	243.11	2.76
ry	20	500	487.14	2.57
ry	50	7000	6847.96	2.17
ry	200	2500	2479.99	0.8
ry	300	500	496.83	0.64
rz	20	7000	7316.44	4.52
rz	50	6000	6000.51	0.01
rz	60	500	499.56	0.01
rz	100	6000	5633.14	6.11
rz	200	500	490.05	1.99
rz	300	200	194.28	2.86

**Table 5:** Muti-frequency acceleration test.

Frequency		Tx (mg)	Ty (mg)	Tz (mg)	Rx (mrad/s^2^)	Ry (mrad/s^2^)	Rz (mrad/s^2^)
20 Hz	Target acceleration	3	3	3	300	500	600
	Actul acceleration	2.91	2.99	2.97	295.7	482.8	586.6
	Magnitude error (%)	3.00	0.33	1.00	1.43	3.44	2.23
150 Hz	Target acceleration	3	2	1	350	400	450
	Actul acceleration	3.02	2.04	0.99	344.0	402.2	452.8
	Magnitude error (%)	0.67	2.00	1.00	1.71	0.55	0.62
300 Hz	Target acceleration	1	2	3	400	400	400
	Actul acceleration	0.98	2.07	3.03	400.9	395.6	403.1
	Magnitude error (%)	2.00	3.50	1.00	0.23	1.10	0.78

## Data Availability

Data might be available from the corresponding author upon reasonable request.
